# Equine-assisted biographical work (EABW) with individuals in the second half of life: study protocol of a multicentre randomised controlled trial

**DOI:** 10.1186/s13063-020-04784-3

**Published:** 2020-10-15

**Authors:** Julia Schmidt, Andrea Wartenberg-Demand, Simon Forstmeier

**Affiliations:** 1 Reittherapie Wittgenstein, Pulvermühle 3, 57319 Bad Berleburg, Germany; 2 Developmental Psychology and Clinical Psychology of the Lifespan, Faculty II, University of Siegen, Adolf-Reichwein-Str. 2a, 57068 Siegen, Germany; 3 Schrecksbach, Germany

**Keywords:** Equine-assisted intervention, Equine-assisted therapy, Biographical work, Reminiscence, Life review, Second half of life, Subclinical depression, Prevention, Randomised controlled study

## Abstract

**Background:**

Equine-assisted therapy is more often practiced with children and adolescents than with the elderly, although individuals in the second half of life could also profit from it. This group, from the age of 50, is characterised by increasing emotional, social, health-related and cognitive changes; a critical life event, such as a neurological illness or loss of a family member, can increase the likelihood of subclinical depression. Individuals who exhibit depressive symptoms not necessarily diagnosed with a major depression may suffer from relevant losses of quality of life (e.g. sleep disorders, memory disorders, feelings of guilt, hopelessness). Despite the fact that the various healthcare systems are in general more frequently used, such individuals often do not receive adequate therapy. The processing of one’s biography (reminiscence) is an elementary component of most psychotherapy approaches and has been demonstrated to treat and prevent the development of major depression. In this study, equine-assisted biographical work (EABW), a combination of equine-assisted therapy and biographical work, will be applied with individuals with subclinical depression in the second half of their life.

**Methods:**

This is a multicentre, prospective, randomised, controlled and open phase III study in enrolling participants with subclinical depression. The aim of the study is to evaluate whether a preventive, equine-assisted, age-specific treatment combining elements of equine-assisted intervention with those of biographical work offers better treatment potentials in comparison to a control group with no intervention. Study participants in the intervention group will receive weekly equine-assisted biographical work over a period of 8 weeks. The primary endpoint is the change in Beck Depression Inventory-II (BDI-II) in a pre-post comparison. Secondary endpoints include other health-related questionnaires including quality of life, reminiscence functions and anxiety.

**Discussion:**

The present study is the first randomised study examining the efficacy of biographical work with a horse and has the potential to establish an empirically based treatment for individuals in the second half of life and improving the symptoms of subclinical depression.

**Trial registration:**

German Clinical Trials Register DRKS00017010. Registered on 01 April 2019

## Background

### Equine-assisted therapy

The horse as a medium in human interaction is used in various social, pedagogical, therapeutic and medical areas due to its many and varied factors of influence [1]. The generic term “equine-assisted interventions” includes, for example, hippotherapy (physiotherapeutic treatment with the horse) and equine-assisted therapy (EAT), which will be explained further below. These approaches differ from each other in their methodology, aims and the basic training of the therapist [2].

EAT is used in combination with psychological settings. The primary target group of this specific intervention are children and adolescents who have a holistic need for therapy [3, 4]. Case studies with adults discussed in the literature are predominantly EAT for the treatment of a diagnosed disorder, whereas preventive approaches receive little attention [5]. Older adults (i.e. aged 65 and older), in contrast, are for the most part left out when it comes to equine-assisted therapy, and only a few study results concerning this group of people are available to date [6].

EAT has a variety of different aims, such as strengthening of self-esteem, strengthening of self-confidence, support of self-efficacy, managing and reducing fears, support of relaxation, identifying resources, improvement in problem solving, support for personal development and improvement of the body sensation.

Well-known studies corroborate the positive effect of equine-assisted interventions on physical development and ability [7] as well as the stabilisation of psychological well-being [8, 9]. In a meta-analysis carried out by Selby and Smith-Osborne, 14 out of 103 studies were selected to represent the biopsychosocial effects of horses on humans [1]. One pilot study mentioned in this meta-analysis should be emphasised which showed statistically significant results for depression syndromes (*p* = .001) for subjects participating in EAT [1, 10].

Although an increasing number of studies have been carried out in the field in recent years, there are only a few significant results confirming the effectiveness of this complementary form of therapy. In most cases, the results are not valid because the studies were often carried out with too small a sample size or without a control group [11].

### Biographical work

Biographical work is assigned as an independent method to the umbrella term of life review and is used in various social, educational, nursing, medical and therapeutic age and work contexts [12]. From among the various definitions of biographical work, it can be summarised that this structured form of self-reflection of one’s life serves to understand the present and to develop resources and capabilities using the knowledge gained in this way for shaping the future [13]. The use of different methods (e.g. body and sense methods, timeline work, laying out the lifeline) can trigger processes of memory [14].

The development theory put forward by Erik H. Erikson is taken as the basis for biographical work [15]. Erikson describes human development as a life-long process taking place in eight phases—from infancy to late adulthood [16, 17].

The main targets of the biographical work are as follows: recall of autobiographical memory contents, raising awareness, recognising own resources, increase of self-confidence, support in finding identity, development of a sense of coherence, increase of the well-being, improving the quality of life and positive life balance.

In principle, biographical work can be used both preventively and to treat existing mental and physical illnesses. Studies show significant positive effects in the structured use of biographical work to improve general well-being, in depression [18–20] and in people with dementia [21, 22]. The positive effect of life review in a therapeutic context (reminiscence therapy) was confirmed in a meta-analysis by Pinquart and Forstmeier [23]. They found a large effect size with regard to depressive symptoms for life review therapy in older people with a clinical depression (*g* = 1.09) and a moderate effect size for the pooled sample of all studies (*g* = 0.57) [23].

### Equine-assisted biographical work

Equine-assisted biographical work (EABW; German: Pferdegestützte Biografiearbeit—Erwachsene, PBA-E) is an integration of equine-assisted therapy into biographical work.

The recently developed concept, which consists of elements of equine-assisted intervention [24] and biographical work or life review [15], has been successfully investigated in various case studiesstudies (Schmidt J: Pferdegestützte Biografiearbeit mit Menschen in der zweiten Lebenshälfte, unpublished). Using the horse as a medium, biographically relevant topics are dealt in a structured way and the different phases of life from childhood to adulthood are considered.

The horse is thus taken in its natural environment and integrated into a process of structured self-reflection, whereby the individual stages of human development can be highlighted and the resulting experiences learned can be used for resource-oriented future design.

Despite the increased number of older adults, especially preventive approaches for older people have received little attention both in equine-assisted prevention and practice to date, despite the fact that older individuals may be able to look back on a joint development history with the horse as a working and farm animal. In equine-assisted therapies, the patient is not expected to have any experience in working with horses. In addition, the focus is not on riding, but on equine-assisted work from the ground.

### Older persons and medical condition

The phase of life from the age of 50 onwards is often marked by significant emotional, social, sensory, motor and cognitive changes. In addition, people in this age group often experience critical life events, e.g. the loss of loved ones, relationship problems, changes in the workplace, poverty and occurrences of diseases [25]. These factors are even more important in view of the ongoing demographic change. These burdens can contribute to the development of subclinical depression, i.e. depressive symptoms manifest themselves, but are not so severe as to justify the diagnosis of clinically relevant major depression. If major depression is defined according to the DSM-V, the person must be experiencing five or more symptoms (e.g. significant weight loss, fatigue or loss of energy nearly every day, recurrent thoughts of death) during the same 2-week period and at least one of the symptoms should be either (1) depressed mood or (2) loss of interest or pleasure [26]. However, subclinical depression is very often associated with a poorer quality of life and greater need for assistance from the healthcare system [27, 28]. In addition, (subclinical) depression can be difficult to diagnose. Affected individuals might contact healthcare providers for various somatic symptoms or complaints before final diagnosis is known [29]. Previous studies in subclinical depression with psychological treatments such as cognitive-behavioural therapy (CBT) have shown moderate effects in treating such symptoms [30].

### Rationale for the current study

At this point, EABW can be applied to individuals who may do not have access to psychotherapeutic treatment due to missing treatment options. EABW could be a measure to support those individuals. In addition, EABW has the potential to motivate those that are not inclined to accept psychotherapy.

Due to the low level of knowledge in this field of research, a pilot study has been undertaken (Schmidt J: Pferdegestützte Biografiearbeit mit Menschen in der zweiten Lebenshälfte, unpublished). The concept was developed using a descriptive questionnaire survey among therapists (*N* = 100). Additionally, a case study (*N* = 2) on EABW with individuals in the second half of life was carried out. In an uncontrolled pretest-posttest design, the effects were measured using a clinical questionnaire battery. The measured positive effects of the individual case study indicate that EABW contributes to a measurable stabilisation of psychological well-being [31] and served as a basis to design this multicentre, prospective, randomised, controlled phase III study.

### Study objectives

The aim of this multicentre, prospective, randomised, controlled and open phase III study is to evaluate whether a preventive, equine-assisted, age-specific treatment of study participants with subclinical depression, combining elements of equine-assisted intervention with those of biographical work, offers better treatment potentials in comparison to a control group with no intervention.

## Methods/design

### Design

This is a multicentre, prospective, randomised, controlled and opened phase III study which includes participants with symptoms of subclinical depression. Those will be randomly assigned to either the intervention or the control group.

Study participants in the intervention group will receive weekly EABW sessions over a period of 8 weeks. According to the study protocol, weekly visits may only take place in a time window of more or less than 3 days. Participants in the control group will undergo no intervention. This control group is chosen because it represents the standard since individuals with subclinical depression seldomly undergo a treatment. Nonetheless, participants in the control group will be offered a minimal intervention consisting of 3 units after the follow-up period. Follow-up assessments will take place 3 months after the intervention.

The primary endpoint is the change in Beck Depression Inventory-II (BDI-II) in a pre-post comparison that will be taken directly before the first and after the last intervention.

The revised version of the Beck Depression Inventory is a self-assessment questionnaire that is used to clarify and diagnose the course of depression. The questionnaire consists of a total of 21 items (e.g. “feelings of guilt”, “crying”, “loss of interest”). The study participant can select four possible answers from 19 items that describe their state of mind within the last 14 days up to the present (e.g. “sadness”: (0) “I am so sad”, (1) “I am often sad”, (2) I am sad all the time, (3) “I am so sad or unhappy that I cannot stand it”). Two items (“change in sleeping habits”, “Change in appetite”) provide seven possible answers by increasing or decreasing the symptoms. Limit values for the BDI-II are as follows: 0–8: no depression, 9–13: minimal depression, 14–19: mild depression, 20–28: moderate depression and 29–63: severe depression [32].

The German BDI-II demonstrates good reliability and validity in clinical and nonclinical samples [33, 34].

Secondary endpoints include other health-related questionnaires (e.g. quality of life, reminiscence and anxiety). Study participants are seen by the therapist at the regular basis. Relevant is the comparison between baseline and week 8 (primary endpoint). We consider further information in the text as too extensive, but they are available in the figure (SPIRIT schedule).

Due to the use of the horse, neither the therapist nor the study participant can be blinded. While the investigator who is responsible for the randomisation is not blind for group assignment, the statisticians who are responsible for statistical analysis will be blind.

### Study sites

Participants will be recruited at 13 locations in Germany. The list of study sites and all relevant documents will be managed by the study coordinator and included in the trial master file. The therapist at each site is the responsible contact person and must be familiar with all the guidelines of the professional association for equine-assisted intervention in Germany (www.berufsverband-pi.de) and the guidelines “Quality assurance for equine-assisted interventions” [35, 36].

The following criteria must be fulfilled to qualify as a therapist:

- Basic educational, psychological, therapeutic or medical profession
- Appropriate equestrian qualification (riding badge and lunging badge)
- Recognised further training in the field of equine-assisted intervention
- Participation in a 1-day training course on EABW and conducting a study according to the International Conference on Harmonisation-Good Clinical Practice (ICH-GCP) guidelines

The central medium in EABW is the specially trained horse (which undergo a training according to conventional riding therapy or riding pedagogy); the consideration of animal welfare is therefore essential for this study. Only physically and mentally healthy animals are used. Each therapist has to be responsible for keeping the horse healthy according to animal protection law, and no additional veterinary care is provided by the sponsor. It is known that all therapists are members of the professional association for equine-assisted intervention in Germany; therefore, a quality assurance is given, also with regard to the well-being of the horses. The sites will be inspected during regular monitoring visits by a well-trained and educated monitor.

The essential building blocks of horse training include a solid basic training in riding, the use of various leading and lunging techniques, the use of classical ground work and free work, accustoming the horse to different materials, a comprehensive relaxation training and a balance training [37, 38].

In order to be able to ensure a high-quality intervention, the following criteria will be examined before a site is included in the study: horse husbandry, feeding, movement areas, constitution of the therapy horses, stables, horse training and education, riding facility and ancillary rooms and safety protocols and facilities.

### Recruitment

Each study centre is responsible for recruiting participants. The therapists will receive materials for the recruitment of study participants that have been approved by the Ethics Committee. In the meanwhile, active advertisements for study participation will be circulated, e.g. in the community and at medical practices.

### Participants

The following eligibility criteria were selected to include participants of either sex with subclinical depression. We decided to use the Beck Depression Inventory-II (BDI-II) to define subclinical depression and the Structured Clinical Interview for DSM-5® Disorders—Clinical Version (SCID-5-CV) to exclude major depression. The following inclusion and exclusion criteria are reviewed by the therapist.

### Inclusion criteria

1. Age ≥ 50
2. Symptoms of subclinical depression (BDI-II ≥ 9)
3. Sufficient physical and mental resilience
4. Adequate language skills
5. Written informed consent

### Exclusion criteria

1. Equinophobia
2. Severe horse hair allergy
3. Diagnosis of major depression based on SCID-5-CV
4. Acute suicidal tendency
5. Psychotic disorders known from anamnesis
6. Known dementia
7. Known severe systemic diseases (e.g. cancer and Parkinson’s disease)
8. Intake of chemical or herbal antidepressants
9. Participation in psychotherapeutic treatment 4 weeks before or during the study (including follow-up)
10. Participation in another study within the last 30 days before inclusion
11. Employment by the therapist
12. Familial relation to the therapist

### Withdrawal criteria

Participation in the study is optional, and there are no negative consequences for refusing participation or dropping out. In order to minimise discontinuation rates, the participants will be informed in detail about the study in written and oral form in advance. The therapist, in consultation with the study coordinator, may decide if it is necessary to discontinue the study for a specific study participant. The reason for termination must be documented in a case report form (CRF).

The study participant may withdraw or be excluded from the study if:

- The written informed consent is revoked
- A drug treatment with antidepressants is indicated
- Any UEs, laboratory abnormalities and medical or psychological conditions would be detrimental or harmful to the study participant if the intervention were continued
- Progression or occurrence of a disease that requires discontinuation of the study
- Significant protocol deviations that require termination of the study
- An exclusion criterion occurs subsequently
- A lack of compliance is apparent
- The contact to the study participant is lost
- It is a request of the study participant

Participants could inform their general practitioner based on an information letter, which they get from the therapist, about the study participation. The accompanying medication, which is indicated due to other basic illnesses, can be further taken by the study participant. With study inclusion, concomitant medication will be documented. If after inclusion in the study additional therapies/treatments are necessary, these are to be communicated to the therapist and it is to be decided whether the study can be continued. If possible, the existing concomitant medication should be continued unchanged during the study. No chemical or herbal antidepressants should be taken during the study. If this is medically necessary or accidentally done, it must be documented as a protocol violation. In addition, no psychotherapeutic treatment should be given during the study.

### Informed consent

The study participants will be informed about the study in accordance with GCP and ethical requirements. The study, its objectives, potential benefits and risks and its consequences will be discussed in detail with the study participants orally by the therapist. Sufficient time is to be allowed to read the written information and ask questions. The study participants may decide not to participate in the study at any time without giving reasons and must not be led to expect any adverse consequences.

The study participant must sign and personally date the informed consent form for participation in the study before any study-related procedures are performed. Persons who cannot give their informed consent will not be included in the study. The written informed consent form is then to be filed and retained by the therapist at the trial site. A duplicate of the signed and dated written informed consent form must be given to the study participant.

### Randomisation

After giving their informed consent, participants will undergo a screening visit, after which the therapist will send the screening documents in encrypted form to the study coordinator. The coordinator will then check the documents for completeness in advance and forwards them to the study director for randomisation. Participants need to meet all requirements for participation in the study in order to be randomised. The allocation ratio for randomisation into either the “intervention group” or the “control group” is 1:1. A randomisation list was created using the software RandList (http://randomisation.eu) before the start of the study. The therapist shall receive written confirmation of the group to which the study participants have been assigned. The pre-test survey begins at the earliest on the following day, at the latest 30 days after randomisation.

### Interventions

Study participants assigned to the intervention group will receive 8 sessions of EABW (90 min each) for approximately 8 weeks. Immediately before the first and after the last intervention, they will be asked about their psychological and health status using a battery of questionnaires. Before and after the interventions, short questionnaires will be applied in order to find out the current psychological state of the study participants. Three months after the last intervention, a follow-up survey will be carried out.

Study participants assigned to the control group will receive no intervention. The same battery of questionnaires will be applied at randomisation and 8 weeks later. After 3 months, the participants complete the follow-up test and may receive 3 minimal interventions of EABW. The first two sessions are scheduled to last 45 min; the last session will last 90 min. Short questionnaires will also be collected before and after the intervention.

Study participants will be regularly seen by the therapist. After each visit, a date for the next visit will be agreed upon. The study participants will be contacted before the next visit in order to guarantee compliance with the protocol. They are informed about the importance of following the exact protocol. It will be avoided to modify the intervention; however, if there is a need to modify the intervention, this will be discussed with the steering committee case by case. Changes to the protocol will be documented as minor or major protocol deviations. Furthermore, regular phone calls will be held with the therapists. If necessary, re-trainings will be performed. The therapists have received audio files on the performance of each intervention.

The interventions within EABW follow a standardised concept (Table 1), which the therapist must comply with.

**Table 1:** Description of sessions

No.	Unit	Focus	Topic
1	Childhood I	Familiarisation with habitat	- Exploration of the horse’s habitat - Triggering of childhood memories - Possible use of nostalgic objects
2	Childhood II	Relationship work	- Relationship building with the horse through body-oriented work - Exploration of the horse’s body - Use of a massage technique - Preparation of the lifeline part 1
3	Adolescence I	Constellation task	- Demonstration of relationships with the help of the horse - Use of horse figures as representatives for family relationships
4	Adolescence II	Communication	- Observing and naming the non-verbal communication of horses - Naming of characteristics of difficult human communication - Preparation of the lifeline part 2
5	Young adulthood	Self-assertion training	- Experiences and dealing with stress - Guiding rope work - Positioning
6	Adulthood I	Parcourse work	- Experiencing and accepting challenges - Dealing with obstacles, formulation of objectives
7	Adulthood II	Mindfulness training	- Recognising and naming one’s own needs - Preparation of the lifeline part 3
8	Integration	Gain of knowledge	- Collecting findings from the previous units - Response to open questions - Review of the complete lifeline

### Statistical analysis

All statistical methods used for the final analysis are described in the study protocol and were detailed in the statistical analysis plan.

The primary target value is the BDI-II for post-testing. The primary analysis is calculated by the full analysis set (FAS), and additional analyses are calculated by the per-protocol set (PP). In addition, the safety analysis is performed with the safety analysis set (SAS). Missing values will be imputed through the last observed value (last observation carried forward (LOCF)). FAS: All study participants who have been randomised (“as randomised”) received at least one intervention and underwent BDI-II baseline testing. These study participants are included in the analysis regardless of protocol violations and therapy termination.

PP: All randomised trial participants who were treated according to the protocol underwent the study without serious protocol violations and for whom the post-test measurement could be performed.

SAS: All study participants who have received at least one of the eight therapy units.

All statistical evaluations are performed with IBM SPSS Statistics 25 (2017). A two-sided significance level of 5% applies to all tests.

A simple analysis of variance (ANOVA) with repeated measurements (pre-test, post-test) will be performed to compare the mean change in depressive symptoms up to the post-test in both groups (factor). In a supplementary analysis of covariance (ANCOVA), the gender, age and severity of depressive symptoms are statistically controlled as covariates.

A further single factor analysis of variance with repeated measurement (pre-test, post-test, follow-up) and an analysis of covariance are calculated to compare the mean change in depressive symptoms until follow-up in both groups.

A multivariate analysis is used to jointly examine the effects of the intervention on the secondary targets. In this way, correlations between the targets are taken into account. Post hoc analyses are performed after a significant overall effect has been found.

### Methods to prevent bias

The proposed study requires several methods to prevent bias.

#### Selection bias

The randomisation procedure is the gold standard to avoid this bias. Random numbers will therefore be generated in advance. The randomisation procedure will be controlled by comparing the two groups with regard to possible confounders, i.e. age at the time of trauma, gender, educational level, diagnosis, cognitive status and trauma severity.

#### Detection bias

The baseline assessment is done before randomisation to avoid this bias.

#### Performance bias

To counteract this bias, the standardisation procedure (manual, extensive training, documentation of sessions, supervision) is established.

#### Attrition bias

Statistical analysis will include techniques to minimise possible attrition bias, i.e. intention-to-treat analysis and imputing missing data. In case of drop-outs, it is intended to continue data collection whenever possible.

### Handling of missing values and drop-outs

A complete data set is available if all three test times (pre-test, post-test, follow-up) have been performed. A drop-out can occur if the participant (1) does not complete the pre-test, (2) decides after information or after randomisation against participation or (3) starts with the treatment, but discontinues it.

If less than 20% of the items of one self-report instrument are missing, missing values are imputed by the mean of the scale of this participant. If a whole scale is missing, the value will be imputed by the expectation-maximation (EM) algorithm.

### Data handling

There are three types of data that is developed or collected in this study: the EABW manual, the “life book” and the assessment data (pre, post and follow-up). The EABW manual will be published with the aim to give professionals with adequate training a resource.

The life book is a folder that the patient fills with photos, important written (positive and negative) memories from all life phases, helpful thoughts, personal and social strengths, information about the family etc. This life book will remain in the possession of the participants and will not be analysed. It is a by-product of the therapy.

The assessment data (information on symptoms) must be considered very sensitive data. Therefore, the data will be stored in an anonymous form. Storage and backup will be ensured during the project by the sponsor in cooperation with the representative of the IT department of the Centre for Information and Media Technology (ZIMT) of the University of Siegen. The long-term archiving will be done in the data repository of the ZIMT.

The data and all associated documentation will be stored for a minimum of 10 years after the completion of the study, including the follow-up period.

### Sample size

The sample size is based on a calculation with G*Power 3.1 [39]. With a medium effect strength (based on the meta-analysis of Cuijpers et al. [30], on psychotherapy for subclinical depression), *α* = 0.05, a test strength of 1 − *β* = 0.80 and a correlation between repeated measurements of *r* = 0.6, the total sample size is *n* = 42. With an assumed drop-out rate of 20%, the total sample size is *n* = 52 (i.e. 26 persons in each group).

### Primary and secondary endpoints

The primary target parameter is the improvement of subclinical depression after 8 weeks as measured by the BDI-II.

The BDI-II is a self-assessment questionnaire used to examine and diagnose the course of depression. All 21 items (e.g. “feelings of guilt”, “crying”, “loss of interest”) are measured on a 4-point Likert scale, and higher scores represent more severe levels of depression. The questionnaire asks about the mental state of the study participant during the last 14 days including up to the survey date [40].

The secondary target parameters include questionnaires dealing with anxiety, behavioural avoidance, ego integrity, positive and negative spontaneous thoughts, gratitude, general self-efficacy expectation, reminiscence functions, state of health and mood state.

**Fig. 1:**
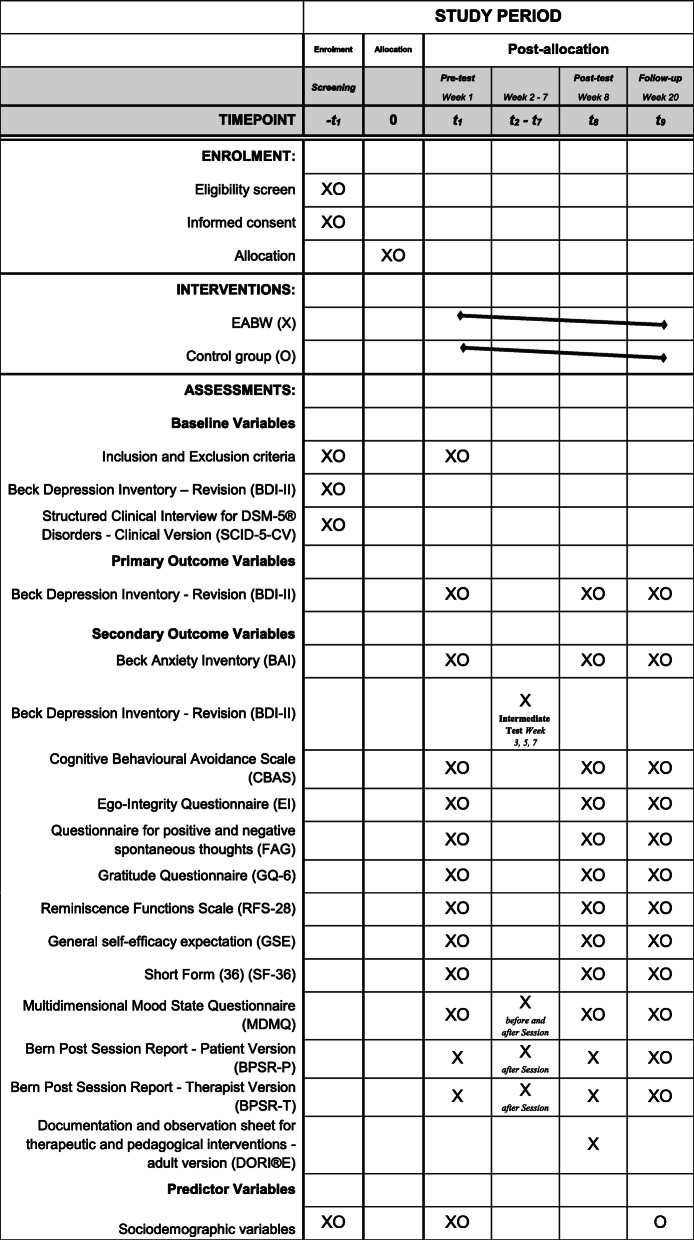
The participant timeline

All questionnaires are processed in printed form. The participant timeline is presented as SPIRIT schedule (Fig. 1).

### Adverse events

Furthermore, the adverse events (AEs) and serious adverse events (SAEs) are recorded and documented as safety parameters. Adverse events are illnesses, signs of disease, symptoms and incidents that occur after inclusion of the participants in the study, but are not necessarily causally linked to the study.

In addition, the severity of the adverse event is defined.

- Non-serious AEs: All such events, whether expected or not, will be recorded.
- Serious AEs: The therapist is obliged to inform the study coordinator within 24 h of the occurrence or suspicion of an SAE. The coordinator reports the SAE to the Study Committee and the Ethics Committee.

The therapist will assess whether the event is:

- Related: There is a comprehensible relationship between participation in the study and AE.
- Not related: There is no traceable relationship between participation in the study and AE.

### Data management and quality assurance

The mental state is measured using a clinical questionnaire battery. In addition to the theoretical background for the development of the respective instrument, comprehensive interpretation guidelines and information on the quality criteria are available for the questionnaires used. In order to check the manageability and applicability of the manual as well as the feasibility of the intervention, the therapist shall receive a separate questionnaire.

All data will be collected in paper-based CRFs. The sponsor will ensure data validation, data verification and compliance through regular monitoring visits according to ICH-GCP. The therapist is obliged to allow the person carrying out the monitoring task (auditor) access to the study material necessary to evaluate the study process. Data protection must be guaranteed according to national law.

After the initiation visit, regular visits will be performed by the auditor followed by a final visit at the end of the study. Additionally, the auditor will maintain contact with the therapist throughout the term of the study.

The data and all appropriate documentation will be kept for a minimum of 15 years after the completion of the study, including the follow-up period.

### Insurance

The study participants are insured by the insurance of the University of Siegen (policy number: H34191780). Additionally, a commuting accident insurance has been taken out (policy number: 50077572427).

## Discussion

“Depression in the elderly is often underestimated” is the title of an article in the *Deutsche Ärzteblatt* (German medical journal), which is based on a survey by *Stiftung Deutsche Depressionshilfe* (German Depression Aid Foundation) [41]. It claims that subclinical depression is a common occurrence among the elderly and that it is associated with a significant health restriction as well as an impairment of quality of life. The prevalence of subclinical depression in adults is at about 10% in Germany, whereas women have a significantly higher prevalence with 11.6% than men with 8.6% [42]. Only 12% of persons > 70 has the opportunity to participate in psychotherapeutic treatment [43]. It is alarming that according to the survey, the suicide rate in older ages is about five times higher than in younger adults [44].

Treatment of subclinical depression is absolutely relevant, due to the increased mortality, the worse quality of life of those affected and the increased strain on the healthcare system resulting in higher costs [45].

To date, little research has been done and no research results are known on equine-assisted therapy (EAT) with adults and seniors suffering from subclinical depression combining biographical work with the horse.

The indications that are treated in equine-assisted interventions in a supportive way are manifold, and more and more studies are being conducted in this area which show positive physical and psychosocial effects [2]. For example, horses are used for the treatment of individuals with post-traumatic stress disorders [8, 46] or autism [47]. However, studies such as these generally still have some limitations (e.g. too small sample size, missing control group, no adequate statistics, no randomisation). The aim of this study is to investigate the effects of equine-assisted biographical work (EABW) in participants with subclinical depression in their second half of life, because there is a limited number of alternative treatment options and preventive measures for individuals in this group are especially important. EABW is not intended to replace conventional psychotherapeutic or drug treatment, but rather help older individuals to identify existing resources by means of a structured review of their own lives with the help of a horse. The idea is to promote self-efficacy and enable a positive life balance.

The concept of EABW is aimed specifically for professionals from the educational or therapeutic field who would like to work preventively with people in the second half of life including horses in individual settings.

Extensive training as well as regular and intensive monitoring and follow-up training will be carried out with the therapists involved, e.g. concerning confidentiality in dealing with participation data. Furthermore, telephone conferences will be held and audio files made available for each intervention to strengthen compliance and the quality of the study.

The sample size in the present study is considered adequate to allow for a demonstration of statistical superiority of equine-assisted intervention.

The primary endpoint concerning the study is taken by a validated questionnaire. This constitutes a patient-reported outcome measure not influence by the therapist.

The design of the study is only limited since a placebo control is not possible. Due to the use of the horse, neither the investigator nor the study participant can be blinded.

In order to implement equine-assisted interventions, it seems to be necessary for therapists to receive equipment that is intended for individuals from the second half of life onwards and their handling of the horse. Therefore, it is planned that the manual on EABW with individuals in the second half of life will be published and that training courses for professionals in equine interventions will be offered. In view of the demographic change in society, EABW offers an opportunity to expand the range of equine-assisted interventions offered by professionals in the long term.

## Trial status

Based on protocol version 2.0 (date 22 March 2019). The trial is in the recruiting phase at the time of manuscript submission. The first study participant was recruited in June 2019, and it is planned that the last participants will join by July 2020.

## Data Availability

The data from this study will be published in peer-reviewed journals. After the publication of the results, the dataset which will be used and/or analysed in the current study as well as statistical codes will be available from the corresponding author on reasonable request. The trial registry will be updated if protocol modifications are made.

## Abbreviations

AE: Adverse event
ANCOVA: Analysis of covariance
ANOVA: Analysis of variance
BAI: Beck Anxiety Inventory
BDI-II: Beck Depression Inventory—revision
BPSR-P: Bern Post Session Report—patient version
BPSR-T: Bern Post Session Report—therapist version
CBAS: Cognitive Behavioural Avoidance Scale
CBT: Cognitive-behavioural therapy
CRF: Case report form
DORI®E: Documentation and observation sheet for therapeutic and pedagogical interventions (adult version)
DoH: Declaration of Helsinki
DSM-V: Diagnostic and Statistical Manual of Mental Disorders
EAT: Equine-assisted therapy
EABW: Equine-assisted biographical work
EI: Ego-integrity questionnaire
FAG: Questionnaire for positive and negative spontaneous thoughts
FAS: Full analysis set
GCP: Good Clinical Practice
GQ-6: Gratitude Questionnaire
GREAT: German Research Center for Equine Assisted Therapy
GSE: General self-efficacy expectation
ICH: International Conference on Harmonisation
MDMQ: Multidimensional Mood State Questionnaire
PBA-E: Pferdegestützte Biografiearbeit—Erwachsene
PP: Per-protocol set
RFS-28: Reminiscence Functions Scale
SAE: Serious adverse event
SAS: Safety analysis set
SCID-5-CV: Structured Clinical Interview for DSM-5® Disorders—Clinical Version
SF-36: Short Form (36)
ZIMT: Centre for Information and Media Technology
